# HOW TO SUCCESSFULLY NAVIGATE A REVISE-AND-RESUBMIT DECISION AND HANDLE REJECTIONS

**DOI:** 10.1093/geroni/igz038.822

**Published:** 2019-11-08

**Authors:** Deborah Carr

**Affiliations:** Boston University, Boston, Massachusetts, United States

## Abstract

Anyone who has ever submitted a manuscript to a peer-review journal has experienced rejection. Rejection can be demoralizing, for emerging and senior scholars alike, but is especially so for those on the job market and tenure-track. This presentation will explain the manuscript review process and suggest strategies for addressing decisions that include: (1) rejection without peer review (desk reject); (2) revise and resubmit invitations; and (3) rejection after peer review. Fixable problems (e.g., editorial, conceptual, minor methodological issues) will be distinguished from non-fixable problems (e.g., small sample size, flawed design). Advice will focus on how to successfully revise and resubmit a manuscript; how to write an effective revision memo; how to develop a “plan B” for a rejected article; and how to manage the emotional sting of rejection.

